# Midwives’ views towards women using mHealth and eHealth to self-monitor their pregnancy: A systematic review of the literature

**DOI:** 10.18332/ejm/126625

**Published:** 2020-09-17

**Authors:** Michelle Vickery, Edwin van Teijlingen, Vanora Hundley, Gary B Smith, Susan Way, Greta Westwood

**Affiliations:** 1 Department of Midwifery & Health Sciences, Faculty of Health and Social Sciences, Bournemouth University, Bournemouth, United Kingdom; 2 Florence Nightingale Foundation, London, United Kingdom

**Keywords:** women, midwives, perceptions, apps, mHealth, eHealth

## Abstract

**INTRODUCTION:**

There are many mobile telephone apps to help women self-monitor aspects of pregnancy and maternal health. This literature review aims to understand midwives’ perspectives on women self-monitoring their pregnancy using eHealth and mHealth, and establish gaps in research.

**METHODS:**

MEDLINE, PubMed, Scopus, CINAHL and PsycINFO were systematically searched on midwifery, eHealth/mHealth and perspectives. Qualitative, quantitative and mixed-methods studies published in English were considered for inclusion in the review, without geographical limitations. Relevant articles were critically appraised and narrative synthesis was conducted.

**RESULTS:**

Twelve relevant papers covering midwives’ perspectives of the use of eHealth and mHealth by pregnant women were obtained for inclusion in this review. Seven of these publications focused on midwives’ views of eHealth, and five on their perspectives of mHealth interventions. The studies included demonstrate that midwives generally hold ambivalent views towards the use of eHealth and mHealth technologies in antenatal care. Often, midwives acknowledged the potential benefits of such technologies, such as their ability to modernise antenatal care and to help women make more informed decisions about their pregnancy. However, midwives were quick to point out the risks and limitations of these, such as the accuracy of conveyed information, and negative impacts on the patient-professional relationship.

**CONCLUSIONS:**

Post-COVID-19, where technology is continuously developing, there is a compelling need for studies that investigate the role of eHealth and mHealth in self-monitoring pregnancy, and the consequences this has for pregnant women, health professionals and organisations, as well as midwifery curricula.

## INTRODUCTION

The technological advancements intrinsic to contemporary society offer new ways of self-monitoring and measuring the human body, through mobile and wearable digital devices, and the internet^1^. Subsequently, the clinic has moved beyond the home, into a sphere where geographical location and time do not limit its accessibility^2^. Consequently, many governments have at the heart of their health policies and strategies self-management by citizens supported by digital data and technology. Self-monitoring may be defined broadly as the efforts made by people to establish and achieve higher goals, by monitoring their behaviour and evaluating their performance^3^. This may also involve people altering their thoughts, feelings, actions and desires^4^, putting them in a position whereby they are an active agent and decision-maker in their life^5^. From a health perspective, this is a key component to successful behaviour change, and may involve the individual observing and recording their eating and exercise behaviours. Examples of innovative technology-enabled care include smartphone apps to facilitate self-management of conditions such as diabetes, ‘health monitors’ as incorporated in smart watches and other devices such as tremor spoon for people with severe tremors such as those with Parkinson’s disease^6^.

The mechanisms used to do this, such as the mobile and wearable digital devices, and the internet, can be categorised into eHealth and mHealth. eHealth, an emerging field of interest to public health^7^, is defined by the World Health Organization^8^ as the use of information and communication technology (ICT) for health. Eysenbach^7^ expands on this, by suggesting the term not only includes the internet and related technologies that deliver health services, but also a state-of-mind that characterises the interconnected commitment possessed by contemporary society to improve healthcare on a local and global level^7^. mHealth is defined as a component of eHealth, which specifically uses mobile communication technology, personal digital assistants, patient monitoring devices and other wireless devices for the delivery of health information and services^9,10^.

Technological advancements lead some to argue that self-monitoring in health is set to increase^11^, including in pregnancy, due to developments such as the smartphone^12^. For example, many thousands of health-related mobile applications are available to the public^1^ and are commonly used by pregnant women as an important information source^13,14^. Hybrid forms exist whereby self-monitoring occurs but under professional control, for example some hospitals now have women monitoring their own blood pressure in pregnancy, but using calibrated monitors that are given to women^6^. Some professionals acknowledge that self-monitoring in pregnancy can lead to women feeling more in control over their decisions, as it gives them a better knowledge of their body^2,14-16^. However, others are quick to express concerns relating to this, due to its tendency to lead to inaccurate results^17^, pregnant women having too much confidence in the unreliable information they source using these mechanisms^18-20^, and the detrimental effects this has for the woman-midwife relationship^20,21^. From the literature it is unclear to what extent this relates to the general notion of many people not liking change or to perhaps a generation gap in familiarity with ICT between older midwives and younger pregnant women.

A literature review by van den Heuvel et al.^14^ also identified serious challenges to the use of eHealth in pregnancy, such as issues surrounding privacy, liability and costs, and a lack of evidence surrounding its effectiveness in pregnancy. Of the 71 articles included in their review, most were published after 2013 indicating an innovative type of care. Traditionally, midwives, obstetricians and general practitioners were the main providers of pregnancy-related information^22^, but technological advancements have revolutionised pregnancy and changed the way it is practiced, making it an embodied project that encompasses digital health, new devices, the internet and responsible bio-citizenship^12^. Pregnancy is one of the most significant changes a woman can experience, and technology is used as a coping mechanism by many to understand the confusing, exciting and frightening changes they encounter^23^. Considering this, the current literature review aims to: 1) understand midwives’ perspectives on women self-monitoring their pregnancy using eHealth and mHealth; and 2) to establish any gaps within the research.

## METHODS

### Study design and search strategy

This review included qualitative, quantitative and mixed methods studies, which were published in peer reviewed journals that discussed the concepts identified using the Population, Exposure, Outcome (PEO) framework (Table 1). A robust search strategy was developed using appropriate Medical Subject Headings (MeSH) and associated synonyms relating to the concepts identified using the PEO framework. As many synonyms as possible were included to ensure that all potentially useful articles were included.

**Table 1:** Population, exposure, outcome (PEO) framework, and medical subject headings (MeSH)*

*Key concepts based on PEO framework*	*Associated synonyms/MeSH*
**Population:** Midwives	Midwife/midwives/midwifery
	Nurse-midwife
	Birth attendant(s)
	Traditional birth attendant(s)
**Exposure:** eHealth and mHealth	eHealth
	mHealth
	telehealth
	telemedicine
	mobile health
	electronic health
	telecommunication(s)
	digital health
	information and communication
	technology/technologies
	ICT
	information technology/technologies
	internet
	mobile technology/technologies
	text message(s)/messaging
**Outcome:** Perspectives (of the population)	Perspective(s)
	View(s)
	Opinion(s)
	Perception(s)/perceive(s)
	Belief(s)/believe Thoughts/think
	Experience(s)
	Attitude(s)

* The three concepts and their synonyms were combined in Boolean phrases, using ‘AND’ and ‘OR’ where necessary in search strings.

Five electronic databases PubMed, Scopus, MEDLINE, PSychINFO and CINAHL were systematically searched up to September 2019 for materials that met the inclusion criteria (Table 2). The three concepts and their synonyms were combined using Boolean phrases, using ‘AND’ and ‘OR’ where necessary. Techniques such as truncation, denoted by an asterisk, and enclosed quotation marks were used when required; the former to search for various spellings and the latter to ensure words appeared together. Database searches were limited to yielding results that included the key concepts and synonyms (Table 1) in their title and abstract only; as Aveyard^24^ argues it is most effective to limit the search to title and abstract only to prevent being overwhelmed by irrelevant results.

**Table 2:** Inclusion and exclusion criteria


**Inclusion criteria**
Midwives’ perspectives of women self-monitoring their pregnancy using eHealth or mHealth
Peer reviewed academic journal articles
Qualitative, quantitative studies, and mixed method studies
No limits on the date of publication or location of the study
English-language publication
Concerned with key concepts identified using the PEO framework
**Exclusion criteria**
Non-peer reviewed articles
Magazine and newspaper articles
Studies on pregnant women’s views of self-monitoring
Non-English-language articles
Not relating to the study topic

Due to a lack of translation resources, only studies published in English were included in the literature review; there were no limitations with regard to year of publication. In order to acquire cross-cultural perspectives where possible, no geographical limitations were applied to the search.

All references retrieved during the systematic search were stored in EndNote and titles and abstracts were screened for eligibility. Articles whose abstracts alluded to the search topic were selected for full-text screening and if relevant, data were extracted and recorded for inclusion in this review. A snowball search strategy was used to identify additional relevant articles from the reference lists of included papers, and their full texts screened to ensure that all potentially useful articles were included.

### Data extraction

A summary of each publication included in this review (Table 3) and the key findings of each study (Table 4) were extracted and recorded in preparation for data synthesis. If the articles concerned the views of other audiences, such as nurses or doctors, only data relating to midwives’ views (including nurse-midwives) were extracted for inclusion in this review.

**Table 3:** Summary of included publications

*Author(s) Year*	*Title of publication*	*Location Year of study*	*Aim of study*	*Method*	*Participants*
eHealth					
Dalton et al.^27^ 2014	‘Who’s afraid?’: Attitudes of midwives to the use of information and communication technologies (ICTs) for delivery of pregnancy-related health information.	Australia 2014	Midwives’ attitudes and experiences of Information Communication Technology use to identify potential causal factors that encourage or inhibit their usage in antenatal care.	Mixed-methods study (semistructured interviews, focus groups, surveys)	19 midwives
Fredriksen et al.^28^ 2018	How do health professionals acknowledge Web-based knowledge in pregnancy consultations?	Norway 2015–2016	To explore how Norway doctors, midwives and physiotherapists manage women’s eHealth literacy and Web-based knowledge in pregnancy	Qualitative study (semi-structured interviews)	13 participants (4 midwives, 4 physiotherapists, 5 GPs)
Johnsen ^18^ 2014	The impact of internet use on the client-professional relationship: A comparative analysis.	Denmark and Norway 2012–2013	To explore how internet use impacts client-professional relationship: midwives compared to other health staff	Qualitative study (semi-structured focus group interviews)	30 health professionals (midwives, nurses and physiotherapists)
Lagan et al.^16^ 2007	Pregnancy problems: answers on the internet?	Northern Ireland 2005	To explore the extent and nature of pregnant women’s use of the internet	Cross-sectional qualitative study (electronic questionnaire)	40 midwives
Lagan et al.^19^ 2011	Web-based survey of midwives’ perceptions of women using the internet and pregnancy: a global phenomenon.	UK, USA, Australia, New Zealand, Ireland, Canada, Netherlands, Germany, Greece, Jordan Mexico 2006	Midwives’ views of internet use in midwifery practice, to elicit extent and nature of pregnant women’s use of internet from a midwifery perspective, and midwives’ views of pregnant women using internet as an information source	Quantitative study (web-based survey)	303 midwives
Wennberg et al.^20^ 2015	A questioned authority meets well-informed pregnant women: a qualitative study examining how midwives perceive their role in dietary counselling.	Sweden 2013	To describe how midwives perceive their role and significance in dietary counselling of pregnant women who use the internet to source information	Mixed-/mono-methods (secondary data analysis from semi-structured telephone interviews and face-to-face interviews	21 midwives
Weston and Anderson^29^ 2014	Internet use in pregnancy.	UK 2013	Value internet use in pregnancy, from viewpoint of: midwives, pregnant and postnatal women	Qualitative study (focus groups and in-depth interviews)	13 midwives, 7 antenatal women and 6 postnatal women
**mHealth**					
Grassl et al.^30^ 2018	A Web-Based Survey Assessing the Attitudes of Health Care Professionals in Germany Toward the Use of Telemedicine in Pregnancy Monitoring: Cross-Sectional Study.	Germany 2017	To investigate the attitudes of health care professionals in obstetrics towards telemedicine.	Quantitative study (web-based survey)	244 health professionals (physicians, midwives, nurses, physician assistants and medical students)
Lanssens et al.^31^ 2019	Midwives’, obstetricians’, and recently delivered mothers’ perceptions of remote monitoring for pre-natal care: Retrospective survey.	Belgium 2016	To investigate the perceptions and experiences of remote monitoring among mothers, midwives and obstetricians.	Quantitative study (online survey)	92 mothers, 52 midwives and 14 obstetricians
Soltani et al.^32^ 2012	Women’s and Midwives’ Perspectives on the Design of a Text Messaging Support for Maternal Obesity Services: An Exploratory Study.	Doncaster, UK 2011	To explore women’s and midwives’ views on the use of mobile technology in supporting obese pregnant women with healthy lifestyle choices	Qualitative study (focus groups using semi-structured interviews)	8 midwives and 6 women
Soltani et al.^33^ 2015	Maternal Obesity Management Using Mobile Technology: A Feasibility Study to Evaluate a Text Messaging Based Complex Intervention during Pregnancy.	Doncaster, UK 2013–2014	To understand the appropriateness of a text messaging based complex intervention for promoting healthy gestational weight gain during pregnancy	Mixed methods (single arm intervention, focus groups and interviews)	14 women and 1 specialist midwife
Willcox et al.^21^ 2015	Views of Women and Health Professionals on mHealth Lifestyle Interventions in Pregnancy: A Qualitative Investigation.	Australia 2013	Women and health staff’s views regarding mHealth sources and interventions to assist women to eat well, be physically active, and gain healthy weight in pregnancy	Qualitative study (focus groups and in-depth, semistructured face-toface interviews)	15 pregnant or postpartum women & 12 health staff (two obstetricians, GPs, midwives, dietitians, physiotherapists, and pharmacists)

**Table 4:** Key findings of included articles

*Author(s) Year*	*Key findings of publication*
Dalton et al.^27^ 2014	Midwives acknowledged that pregnant women are increasingly using the internet and mobile technologies to seek pregnancy related information. Midwives recognised both the potential benefits and possible risks in the use of Information Communication Technologies (ICTs) in the delivery of pregnancy-related health information, but expressed significant concerns around the accuracy of the information available online.
Fredriksen et al.^28^ 2018	All of the participants had experienced pregnant women having web-based knowledge either directly or indirectly Generally, participants were ambivalent towards women using eHealth to source pregnancy related information and were especially sceptical about web forums as they were deemed as misinformative. Many of the participants felt that pregnant women’s eHealth literacy challenged their professional role and authority, subsequently having a negative impact on the patient-professional relationship. Midwives felt it was time consuming to help pregnant women differentiate between accurate and untrustworthy information and that pregnant women using the internet to obtain pregnancy-related information had resulted in requests for extra consultations due to anxieties and worries around the information they had sought. Midwives were more net friendly in their clinical practice than other participants, and distributed links to trustworthy online information, encouraged against web forums and encouraged critical thinking by their patients when appraising the quality of the information they had sourced.
Grassl et al.^30^ 2018	There is an ambivalent attitude towards the use of telemedicine amongst healthcare professionals. Midwives felt that an app which pregnant women could consult when feeling unwell or experiencing unfamiliar symptoms that would give advice or advise them to see a doctor would lead to unnecessary emergency consultations increasing their workload. 72.6% of participants had doubts about mHealth developments in antenatal care and few would recommend this to their patients.
Johnsen^18^ 2014 information.	Overall, midwives were mostly negative about the use of the internet by pregnant women to gather pregnancy-related information Midwives raised concerns around the reliability and accuracy of information being acquired, and felt this caused pregnant women to rely upon the midwives to authenticate this information. Midwives felt the volume of information caused information clutter, which they often had to clear. Midwives felt their knowledge and experience was undermined by information gathered by pregnant women in chatrooms, especially when they valued the information from other pregnant women more than that of the midwife. Although pregnant women were extremely updated on health information retrieved from the internet, midwives felt the women were extremely unlikely to act upon this without consulting them first.
Lagan et al.^19^ 2011	Midwives recognised that pregnant women are increasingly using the internet to gather pregnancy related information and just under three-quarters of participants recognised the benefits of this. Despite this 90% of participants expressed significant concerns about the accuracy of information available on the internet. 89% of midwives perceived pregnant women to be increasingly using the internet. This was noted in each individual country included in the study, except Jordan. 73% of respondents agreed or strongly agreed that the internet improves the pregnant woman’s knowledge of pregnancy-related health conditions and treatments, and gives them more control over the choices surrounding their pregnancy. In the study years of 2005–2006, 86% of midwives had had experience of women discussing information they had acquired from the internet, with the main sources cited to obtain information being Google and Yahoo.
Lagan et al.^16^ 2007	The results suggested an increased use of the internet to acquire pregnancy-related information. Midwives were positive about the use of the internet by pregnant women, and felt it has the ability to improve healthcare delivery and information dissemination. 69% (n=24) of midwives reported in the last year (2004–2005) a pregnant woman had discussed information with them that they had retrieved from the internet, much of which was obtained from search engines such as Yahoo or Google.
Lanssens et al.^31^ 2019	Although most of the participants had little or no experience with remote monitoring technology, they reported positive perceptions of this, and felt it was not a threat to their everyday work. Remote monitoring was perceived as an important component in the follow up of high-risk pregnancies, with 77% of midwives believing that it improved the care for high risk pregnancies and 80% reporting that it added value to pregnant women.
Soltani et al.^32^ 2012	Although quicker to identify limitations and risks, midwives were generally positive about the use of a text messaging service to support pregnant obese women with making healthy lifestyle choices. Midwives believe the scheme had the ability to modernise, motivate, remind and reduce the sense of isolation amongst pregnant obese women, all of which could effectively help them to make healthy lifestyle choices. Midwives were quick to identify the possibility of the service being offensive and creating pressure or guilt amongst its users. Midwives felt it important to make the service available to all pregnant women, and emphasised the message tone, content, and other forms of supportive mobile technology should be given special attention. The specialist midwife felt positive about the use of a text messaging service to promote healthy gestational weight gain as they were able to build rapport and tailor the messages, but felt negative about the logistics of the service. Relationship building was perceived as a major advantage of the initiative, as the midwife was able to create trust through appointments with the pregnant women to support them alongside the text messaging system and self-monitoring activities. The midwife felt personalised support was beneficial as she could tailor messages to encourage or praise each pregnant woman to support their healthier lifestyle changes, and she could also use the mobile technology to refer them to further support services which made this a holistic intervention. The midwife was negative about the information technology and logistics as the lists of messages to select were too long and the process of selecting them had too many stages meaning it was not as time efficient as it could have been.
Wennberg et al.^20^ 2015	Overall, midwives felt positive about the use of the internet by pregnant women because they believed it made them well-informed, however they were concerned about the accuracy of the information that was being accessed. Midwives felt that women needed professional guidance to interpret this information as they could often be too emotionally oriented, lack rationality, and be too worried to assess the information adequately. Midwives felt that often women had too much confidence in the information they acquired, making them feel like a questioned authority. Midwives often felt less well informed than pregnant on dietary issues as they did not have time to search for the information, leading to feelings of inadequacy. Although they felt listened to, midwives felt unsure of the impact their advice had on the pregnant women’s behaviour.
Weston and Anderson ^29^ 201	Midwives were mostly negative about the use of the internet by pregnant women due to concerns around their inappropriate use of this, and specifically expressed concerns relating to overuse creating anxiety, pregnant women becoming obsessed by internet usage to acquire information, their poor judgement of the information they acquire, and the unrealistic expectations it creates with regards to managing pregnancy. Midwives felt a beneficial aspect of the internet was that it has the ability to empower pregnant women and act as a discussion trigger, however felt it was often used by pregnant women to challenge the midwives’ provision of care and expertise, and that social media and apps are encouraging women to disbelieve midwives’ advice. Midwives were negative about discussion forums as the experiences shared do not apply to all pregnant women, but were positive about pregnant women using the NHS website
Willcox et al.^21^ 2015	Health professionals were generally negative about the use of mHealth, were quick to identify the associated risks and felt that mHealth was detrimental to the patient-professional relationship. Some health professionals felt that the emergence of technology has shifted the control of information to untrusted sources, and away from trusted health professionals and organisations. Many health professionals expressed concerns regarding the medicolegal risks associated with mHealth, such as the harm to the women (harmful information and privacy issues), and the harm to the professional integrity of health professionals and organisations (intellectual property, privacy, legitimacy concerns). However, others acknowledged mHealth was feasible if these risks were addressed. The unfamiliarity with and fear of mHealth meant some health professionals had limited engagement with and understanding of its ability to support antenatal care. Some health professionals expressed concerns regarding the accessibility of mHealth to women who may not have mobile phone access.

### Quality assessment

In order to assess the quality of each article, the Critical Appraisal Skills Programme (CASP) Systematic Review Checklist 2017^25^ was utilised. This technique helps to verify that studies are trustworthy, and subsequently ensures the chosen literature was eligible to include in this review.

### Data synthesis

The characteristics of each study (Table 3) and their key findings (Table 4), were summarised. The relevant articles included in this review were narratively synthesised under two headings: eHealth and mHealth.

## RESULTS

**Figure 1:**
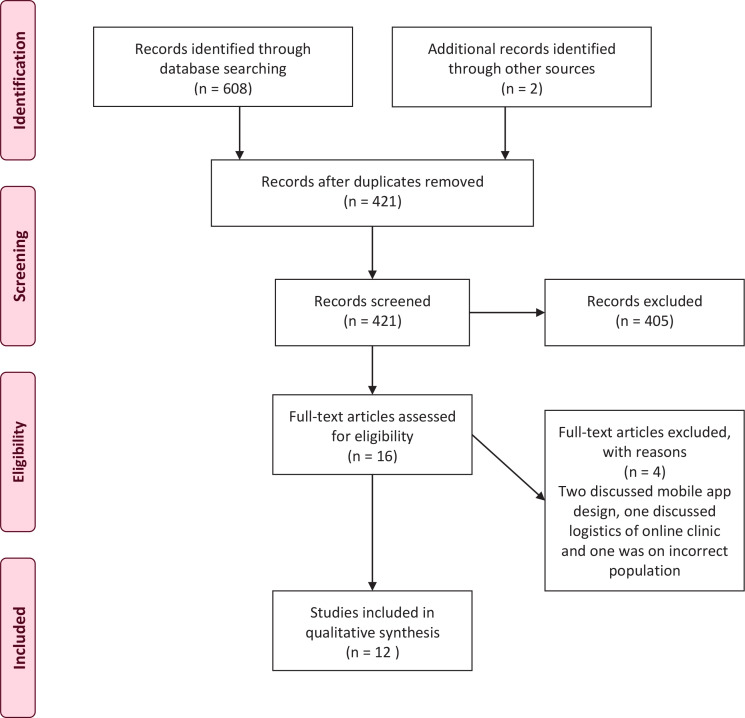
PRISMA flowchart

A total of 610 papers were identified after duplicates were removed, 421 articles remained for title and abstract screening. During this stage, 405 were excluded as they were not relevant to the search topic, leaving 16 publications, which had their full-texts assessed for eligibility. Four out of the 16 articles were excluded with reasons, leaving 12 publications for inclusion. The process for identification, screening, eligibility and inclusion, which underpins this systematic search, is illustrated by the PRISMA flowchart^26^ depicted in Figure 1.

### Studies of the review

The twelve studies included in this review are described in Table 3. All studies were conducted in high-income countries, with the majority of the studies from the UK.

The findings are presented under two broad categories: eHealth, which includes seven of the studies, and mHealth which includes five. Each category has its own subcategories within it, based on themes that were identified throughout the articles. A summary of the key findings of the articles are discussed individually in Table 4.

### eHealth

Seven of the studies focused on midwives’ views of pregnant women using eHealth to acquire pregnancy-related information (Table 3). Participants in all of the studies perceived that there was an increase in internet usage by childbearing women. One international study found that 89% (n=271) of midwives from ten countries perceived that there was an increase in internet usage amongst pregnant women, with 91% in the UK alone considering there was a rise^19^. Throughout the seven studies, which related to midwives’ views of pregnant women’s use of eHealth, three common issues were identified and are discussed in more depth below: Accuracy, Informed choices, and The midwifewoman relationship.

#### Accuracy

Six studies reported that midwives were concerned about the accuracy and reliability of the information that was being accessed by pregnant women on the internet^18-20,27-29^. One study found that 90% of respondents were very or somewhat concerned about the accuracy of information that pregnant women were accessing^19^, whilst another found that midwives were specifically concerned about the quality of information that younger women who ‘lived on the net’ were exposed to^20^. Participants from two of these studies discussed how this information would often create anxiety and unrealistic expectations of pregnancy management amongst the women, and would often result in requests for extra consultations due to concerns about the information they had sought^28,29^. Participants in three of the studies discussed how midwives were often relied on by pregnant women to help them to distinguish between accurate and untrustworthy information they had retrieved by using the internet^18,20,28^. Midwives felt that the information pregnant women retrieved from the internet caused information clutter, which had to be cleared by the midwives^18^, and that helping pregnant women to authenticate information was a time-consuming process^28^. Midwives in three of the studies expressed concerns about the use of web forums to retrieve pregnancy-related information^27-29^ as they were deemed as misinformative sources^28^ containing extreme experiences that do not apply to all pregnant women^29^. Despite concerns about the accuracy of information, midwives in the Australian study by Dalton et al.^27^ failed to consistently inform women about evidence-based websites, however, when they did, they directed their patients to the South Australian Government website. Midwives in the study conducted by Fredriksen et al.^28^ did provide links to trustworthy websites and participants in the study by Weston and Anderson^29^ were positive about the use of the NHS website to retrieve pregnancy-related information. Although the study by Lagan et al.^16^ found that one participant had used PubMed to access pregnancy-related information, Google and Yahoo were found to be predominately used to retrieve this^16,19^.

#### Informed decisions

Four of the studies unanimously agreed that the internet possessed the ability to widely disseminate information making women more knowledgeable about many aspects of their pregnancy, including pregnancy-related health conditions and treatments, helping them to become more actively involved in the decisions relating to their pregnancy^18-20,29^. In one study, midwives felt that pregnancy-related information retrieved from the internet helped to empower pregnant women and acted as a discussion trigger^29^. Lagan et al.^19^ found that 73% of midwives agreed that the internet gave childbearing women more control over the choices surrounding their pregnancy and improved their knowledge of pregnancy-related health conditions and treatments^19^. Participants from two studies agreed that women would not act on the information they acquired on the internet without consulting a midwife first, as they needed help to interpret and authenticate this knowledge^18,20^ and that information prepared pregnant women for their consultations, acting as a source of comparison^18^. Lagan et al.^19^ found that of 86% of midwives who had experience of women discussing internet gathered information in the year 2005–2006, 67% thought this affected how the woman believed their pregnancy should be managed^19^.

#### Midwife-woman relationship

Although participants agreed that the internet helps pregnant women to make more informed decisions, midwives from four of the studies felt that the pregnant women’s use of the internet had negative impacts on the traditional midwife-woman relationship^18,20,28,29^. Participants from these studies stated that women often had too much confidence in the information they were accessing, which resulted in the midwife becoming a questioned authority^20^ whose professional role was undermined by internet gathered information^18,28,29^. Midwives in one study felt their knowledge and experience was especially undermined by the information retrieved by pregnant women from online forums, as they seem to value the experiences of other women more than that of the midwive^18^. Two studies found that that midwives often felt inadequate as the women were often better informed than them on current information and studies, as they had no time to update themselves^20,28^. Midwives felt that the knowledge retrieved by pregnant women from the internet was often used to challenge their provision of care^29^ and that it was creating more demanding healthcare users^28^. Wennberg et al.^20^ found that midwives felt listened to, however, they were unsure of the impact their advice had on the pregnant women’s behaviour^20^. In contrast, Fredriksen et al.^28^ found that midwives felt that pregnant women used information retrieved on the internet to make their decisions prior to their consultations, and so their professional opinion was undervalued. Alongside the internet, Weston and Anderson^29^ identified that midwives believed that social media and mobile apps were encouraging pregnant women to disbelieve their advice, challenging their professional authority.

### mHealth

Five studies focused on the views of midwives on the use of mHealth within pregnancy^21,30-33^. Two of the studies explored midwives’ views on the use of a Short Messaging Service (SMS) to deliver information and support to women throughout their pregnancy^32,33^; one discussed midwives’ perceptions of the use of mHealth to source and deliver pregnancy-related information^21^; one focused on the perceptions of midwives regarding remote monitoring during pregnancy^31^; and the final study aimed to investigate the attitudes of midwives towards a pregnancy-related app^30^. Due to the variation of technology discussed and the differing themes highlighted, results are discussed in more depth below under two categories: Benefits of mHealth, and Limitations and risks of mHealth.

#### Benefits of mHealth

Three studies found that midwives were predominately positive about the use of mHealth within pregnancy^31-33^. Two of these studies explored midwives’ perspectives of a Short Messaging Service (SMS)^32,33^, whilst the other investigated the perceptions of midwives regarding remote monitoring during pregnancy^31^. Both studies by Soltani et al.^32,33^ found that midwives expressed positivity about the SMS schemes, which were designed to support women with gestational weight gain. One study identified the benefits of the SMS initiative to be its ability to modernise antenatal care, motivate and remind pregnant women about their goals of weight management, and decrease their sense of isolation^32^. The specialist midwife in the study by Soltani et al.^33^ also expressed positivity about the scheme, and believed that using an integrated service, which combined appointments with self-monitoring activities and mHealth, created a strong trust between the midwife and the women, which effectively supported their weight management. The specialist midwife in this study felt that her ability to tailor the text messages to each individual woman was extremely beneficial, as this meant she could support and praise each woman according to her requirements and achievements^33^. Furthermore, they could refer them to other support services via the mobile technology, making the intervention holistic^33^. The remaining study, conducted in Belgium by Lanssens et al.^31^, echoed the positive views of midwives regarding mHealth that were identified in the studies by Soltani et al.^32,33^. Lanssens et al.^31^ found that midwives felt remote monitoring of pregnancies complemented their everyday roles, and they perceived it to be an important component in the management of high-risk pregnancies, despite their lack of prior experience with this technology.

#### Limitations and risks of mHealth

Three of the studies found that midwives were quick to identify the limitations and risks of mHealth^21,30,32^. Despite midwives holding predominately positive views of an SMS intervention in the study by Soltani et al.^32^, midwives were quicker to outline its limitations and risks than benefits. They would mentioned, for example, its potential to create offence through messages that may be deemed insensitive by women, its ability to generate feelings of pressure or guilt amongst women, how the impact of the scheme may be influenced by the individual’s mood or motivation, and its potential inaccessibility to some women who may not have phones. Willcox et al.^21^ found that participants were generally negative about the use of mHealth to source and disseminate pregnancy-related information, and quickly identified inherent risks, such as medicolegal ones, including harmful information and privacy issues, and harm to professional integrity of health professionals and organisations, including threats to their intellectual property, and concerns surrounding privacy and legitimacy^21^. In line with the study conducted by Soltani et al.^32^, participants expressed concerns surrounding the potential for mHealth to exclude women who do not have mobile phones^21^. Further, pessimism was expressed by participants who felt that mHealth has had and will continue to have detrimental effects to the patient-professional relationship because it has shifted the control of information from trusted to untrusted sources. However, it was acknowledged by some of the participants that their unfamiliarity with mHealth inhibits their ability to envisage its potential to support antenatal care^21^. Midwives in the study conducted by Grassl et al.^30^ expressed negativity about the use of mHealth, namely an app in pregnancy monitoring, as they felt it would lead to unnecessary emergency consultations increasing their work load. This view coincides with that of participants in the eHealth studies by Fredriksen et al.^28^ and Weston and Anderson^29^, who felt that pregnant women’s use of eHealth often resulted in requests for extra consultations due to anxieties created by the information they had sought.

## DISCUSSION

This systematic review aimed to identify the existing literature available regarding midwives’ perceptions of women self-monitoring their pregnancy using eHealth and mHealth. The review found that the use of eHealth by pregnant women and mHealth interventions in antenatal care were the only forms of self-monitoring that were discussed from the perspectives of midwives. Interestingly these mechanisms were not specifically referred to in publications as forms of self-monitoring, but as means of information seeking and dissemination, and support provision. A total of twelve papers were included, seven explored eHealth and five mHealth.

The review identified unanimity that midwives feel women are increasingly accessing pregnancy-related information through eHealth, whether to acquire knowledge or to help them in making pregnancy-related decisions. Generally, midwives held ambivalent perspectives regarding the use of eHealth by pregnant women, but these can be categorised as more negative than positive. In many of the studies, much of this negativity derived from concerns about the accuracy of the information that women were accessing and using to inform their decisions^18-20,27-29^. Many midwives also expressed much apprehension around the negative impacts that eHealth is having and will continue to have on the traditional midwife-woman relationship, and raised concerns that it undermines their professional role^18,20,28,29^. Such changes in the healthcare provider–service-user relationship and required changes in the professionals’ ways of working are not easy, especially not in the beginning, as recognised by doctors in Portugal and Israel^34^. Whilst a study on eHealth in musculoskeletal models of care suggested the training not only of clinical staff but also administrative support staff^35^.

Although in some studies midwives felt women would not act upon internet-retrieved information without their authentication, as they required help to decipher between accurate and untrustworthy information, others believed internet-acquired information was more highly valued by the women than the information provided by the midwives, making them question professional advice and the midwives’ provision of care^18,20,28,29^. Midwives may even fear the knowledge of pregnant women as it can make them feel inadequate, as they do not have time to update their knowledge and skills^20,28^. It is possible that there is a generation gap, with older midwives being less experienced and less confident in using internet-based technology and information than their younger clients. In the short-term this gap could be filled by specific training on eHealth and mHealth for more established midwives, midwifery leaders, and educators. In the longer term this would probably require the updating of midwives’ job descriptions and midwifery education. Despite the predominant negativity surrounding eHealth, midwives in some studies did express some positivity around the ability of eHealth to make women more knowledgeable, helping them to make informed decisions about their pregnancy^16,18-20,29^.

As with eHealth, midwives held mixed perspectives on the use of mHealth in the self-monitoring of pregnancy. In three studies, participants were quicker to identify the inherent limitations and risks of mHealth^21,30,32^; a trait which one publication noted could originate in their professional code of conduct of doing no harm^32^. This view is concurrent with the study conducted by Willcox et al.^21^, where participants raised medicolegal concerns about harmful information and privacy issues, and harm to professional integrity. However, Willcox et al.^21^ suggest the pessimism expressed in this particular study may have originated in the participants’ unfamiliarity with mHealth. Some suggest this illustrates a need for midwives to be given the opportunity to develop their internet skills^19^ and be taught about the benefits of technology to antenatal care^21^, in order to be able to better collaborate with pregnant women to access verified information^28^. Studies by Soltani et al.^33^ and Lanssens et al.^31^ found that midwives held predominately positive views of mHealth, despite midwives being quick to outline its limitations and risks regarding the logistics of the scheme. Benefits of mHealth interventions were noted as its ability to modernise antenatal care^32^, to create a strong trust between the midwife and the woman^33^, and as a technology that could complement the midwife’s everyday role^31^. More generally, maternity care organisations, health workers, consumer organisations and service users may want to consult national and international advice on digital health such as the WHO 2019^36^ guidelines. Moreover, midwives and their professional organisations should work with the International Confederation of Midwives (ICM) on ways to incorporate eHealth and mHealth in midwifery curricula.

### Strengths and limitations

This review carried out a systematic search of five electronic databases to identify relevant papers: MEDLINE, PubMed, Scopus, CINAHL and PsycINFO. In addition to this, a snowball search strategy was used to identify additional relevant articles from the reference lists of papers, which were selected for inclusion in this review, and their full texts were screened to ensure no potentially relevant articles were excluded. Whilst a reproducible search strategy was used, it is possible that studies indexed elsewhere were not identified and not cited by the included studies.

As part of the inclusion criteria, articles had to be published in English, due to a lack of translation resources. Subsequently, a limitation of this review is that relevant articles that were published or available in any language other than English may have been excluded for this reason. This potentially has implications on the transferability of the findings outlined in this review, to Low- and Middle-Income Countries and countries where English is not the native language. Therefore, it is important to acknowledge that the findings in this review may not accurately reflect midwives who work in other settings outside the UK. Finally, this review was undertaken prior to the occurrence of COVID-19; the public health measures employed in many countries such as lockdown, restrictions in travel, meeting people and social distancing, saw a rapid increase in online health services including online COVID-19 symptoms checkers^37^.

## CONCLUSIONS

It seems inevitable that women will increasingly use easily available online information to help them make pregnancy-related decisions. This review noted that eHealth and mHealth are the only forms of self-monitoring that have been explored from the perspective of midwives in the academic sphere. eHealth is being used increasingly by women to access pregnancy-related information, and mHealth initiatives are beginning to be utilised. However, with only twelve relevant articles identified in this field, there is a compelling need for more research that explores midwives’ perspectives of women self-monitoring their pregnancy, with regard to both eHealth and mHealth, but also more broadly concerning their perspectives of selfmonitoring devices and home-monitoring equipment. In our contemporary society, midwives will have to find new ways to adapt to and accept these changes, whether by educating themselves about the benefits of technology or developing the necessary skills to use this. However, it is important to remember that there are limitations to eHealth and mHealth, including: 1) lack of privacy; 2) issues of liability and costs; and, most of all, 3) lack of evidence about its effectiveness in pregnancy.
